# Are we measuring loneliness in the same way in men and women in the general population and in the older population? Two studies of measurement equivalence

**DOI:** 10.1371/journal.pone.0266167

**Published:** 2022-12-29

**Authors:** Thomas V. Pollet, Alexandra Thompson, Connor Malcolm, Kristofor McCarty, Tamsin K. Saxton, Sam G. B. Roberts

**Affiliations:** 1 Dept. of Psychology, Northumbria University, Newcastle upon Tyne, United Kingdom; 2 Dept. of Psychology, Liverpool John Moores University, Liverpool, United Kingdom; Shenzhen University, CHINA

## Abstract

**Background:**

High levels of loneliness are associated with negative health outcomes and there are several different types of interventions targeted at reducing feelings of loneliness. It is therefore important to accurately measure loneliness. A key unresolved debate in the conceptualisation and measurement of loneliness is whether it has a unidimensional or multidimensional structure. The aim of this study was to examine the dimensional structure of the widely used UCLA Loneliness Scale and establish whether this factorial structure is equivalent in men and women.

**Methods and sample:**

Two online UK-based samples were recruited using Prolific. The participants in Study 1 were 492 adults, selected to be nationally representative by age and gender, whilst the participants in Study 2 were 290 older adults aged over 64. In both studies, participants completed the UCLA Loneliness Scale (Version 3) as part of a larger project.

**Results:**

In both studies, the best fitting model was one with three factors corresponding to ‘Isolation,’ ‘Relational Connectedness,’ and ‘Collective Connectedness.’ A unidimensional single factor model was a substantially worse fit in both studies. In both studies, there were no meaningful differences between men and women in any of the three factors, suggesting measurement invariance across genders.

**Conclusion:**

These results are consistent with previous research in supporting a multidimensional, three factor structure to the UCLA scale, rather than a unidimensional structure. Further, the measurement invariance across genders suggests that the UCLA scale can be used to compare levels of loneliness across men and women. Overall the results suggest that loneliness has different facets and thus future research should consider treating the UCLA loneliness scale as a multidimensional scale, or using other scales which are designed to measure the different aspects of loneliness.

## Introduction

Throughout their evolutionary history, humans have lived in social groups and depended on forming long-term relationships with others for survival [1, 2]. Thus, humans have a basic and universal need to form strong, stable interpersonal relationships with others—a ‘need to belong’ [3]. When this need is unmet and people feel disconnected from others, this lack of meaningful social relationships has a profound impact on physical and mental health [4]. Loneliness is defined as an unpleasant subjective state arising from a mismatch between the quantity and quality of social relationships we have and those we would like to have [5].

A large body of research has demonstrated that high levels of loneliness are associated with negative health outcomes in relation to both morbidity and mortality (reviews in [6–11]). Loneliness also has a key place on the social and political agenda in countries such as the United Kingdom [12], and the pandemic has further exacerbated the need for policy intervention on this front [13]. It is thus important that we can reliably measure loneliness, in order to accurately measure its prevalence over time, in different parts of the population and to evaluate whether interventions to combat loneliness are effective [14, 15].

Over the past five decades, many scales have been developed to measure loneliness, including: the Differential Loneliness Scale [16], the Loneliness Rating Scale [17], the De Jong-Gierveld Loneliness scale [18], and the Social and Emotional Loneliness Scale for Adults (SELSA, [19]). One of the most commonly used measures is the UCLA Loneliness Scale, which has appeared in first [20], second [21] and third [22] versions, and its short form adaptations (e.g., [23–25]). The UK Office for National Statistics has recommended that future UK national surveys of loneliness use three items from the UCLA scale [26]. The scale has been translated into many languages (e.g., Russian: [27]) and validated in many countries (e.g., Italy: [28]; Zimbabwe: [29]).

### UCLA loneliness factor structure: One, two, or three factors?

A key unresolved debate in the conceptualisation and measurement of loneliness is whether it has a unidimensional or multidimensional structure [20–22, 30–32]. From its inception, the UCLA Loneliness Scale was argued to tap into a unidimensional construct [20–22], with deficits in a variety of relationships producing the same underlying state. Indeed, many studies have found evidence for a unidimensional structure (e.g., [33, 34]), or for a unidimensional structure with a subsidiary factor accounting for methodological effects due to wording [35]. Some such studies have used student participants, for example, a sample of over 650 South African students supported a one-factor solution [34]. Yet a one-factor solution is also supported in other samples, such as adolescents (e.g., [36]). Other studies (e.g., [37, 38]) do not conduct factor analyses to establish the factor structure of the UCLA Loneliness Scale, but instead, treat the scale as defining a unitary construct. A synthesis of eighty studies using the UCLA Loneliness Scale as a unidimensional construct revealed an estimate of Cronbach’s *α* of .87 [39]. The size of this estimate depended on four factors: article type (focussing on measurement or not), scale standard deviation, whether a social support network was measured, and sample composition. Interestingly, in terms of sample composition, adolescent samples tended to yield lower reliabilities than non-adolescent samples. However, whether a sample was composed of older adults or not did not influence the reliability estimate.

From its inception, however, the unidimensional nature of the UCLA loneliness scale has been challenged on both theoretical and statistical grounds (e.g., [40, 41]). Studies have argued for two (e.g., [29]), three [42] or even four or five factor solutions (e.g., [23, 43–45]). There are only a minority of papers reporting four and five factor models respectively, so we restrict our review of the literature to two and three factor models. Whilst some argue loneliness is a unitary state [21, 22], other researchers propose that loneliness has two key components: emotional and social isolation (e.g., [32, 46]). Thus, Weiss [32, 41] argued that the need for the emotional security provided by a single ‘attachment figure’ is distinct from the need to be connected to a broader social network, and people can be dissatisfied with one aspect (e.g., lack of a long term romantic partner) without being dissatisfied with the other (e.g., having a good network of friends). In line with this proposition, Zakahi and colleagues [47] argued for a two factor solution. Similarly to Zakahi and colleagues [47], Wilson and colleagues [29] recovered a two-dimensional factor structure in a sample from Zimbabwe. These two factors were labelled as “social other” and “intimate other.” However, Knight and colleagues [48], while recovering a similar factor structure, attributed this to the framing of items as positive or negative. Accordingly, Russell [22] revised the scale (UCLA Loneliness Scale Version 3) and suggested a two-dimensional structure. Using this Version 3 of the UCLA Scale, some studies have found support for the two-factor structure. For example, Ausín and colleagues [49] found support for a two-factor model in a large sample (n > 400) of adults aged 65 or over.

However, other research has argued for a three-factor structure for the UCLA loneliness scale (e.g., [42, 50, 51]). One such three-factor structure is Russell’s model [22], which allocates all items to one factor, and then additionally allocates each item to either a “negative items” factor or to a “positive items” factor. This structure has been supported using confirmatory factor analyses in relation to the UCLA Scale Version 3 [22] in two Turkish samples [52], and in a sample of 300 healthy Iranian adults [53]. Similarly, a sample of over 500 respondents from Argentina [54] supported this model using the second version of the UCLA [21]. Given the range of studies supporting the Russell model [22] model, we attempt to fit this model to our data, below. Other three-factor solutions have also been put forward in relation to the second and third versions of the UCLA, and these more conventionally allocate each item to one factor exclusively. These solutions include McWhirter et al.’s model [50] which named the factors “Intimate Others,” “Social Others,” and “Affiliative Environment”; Boffo and colleagues [28] who named the factors “Isolation,” “Relational Connectedness,” and “‘Trait Loneliness”; and Sancho and colleagues who named the factors “Isolation,” “Trait Loneliness,” and “Social Connectedness” [55]. Most notably, however, the work by Hawkley and colleagues [40] argued for the following three factors: “Isolation,” reflecting feelings of rejection and aloneness; “Relational Connectedness,” corresponding to feelings of familiarity; and “Collective Connectedness,” which deals with feelings of group identification. This model has received support from large-sample studies, including one of over 1,400 Irish adolescents [56], and another that relied on student samples (n > 500) [57]. Contrastingly, a study using participants from Indonesia, Germany, and the United States, did not find the three factor solution to be a good fit in absolute terms [31], although a three factor solution did perform slightly better than a one or two factor solution. Given this range of support, we test this latter three-factor model [40] in our analysis below, together with the unidimensional model as proposed by Russell and colleagues [21, 22].

### Gender differences

Research exploring gender differences in loneliness presents mixed findings, with some research suggesting that women report more loneliness than men (e.g., [58, 59]), some research indicating that men report more loneliness than women (e.g., [22, 60–63]), and yet other research not finding a robust gender difference (e.g., [64]). In addition, much of this research has tended to rely on scales with a unidimensional approach to loneliness, rather than a multidimensional approach (but see [65]). It is important to establish that the scales used yield the same factorial structure for men and women to enable us to make valid comparisons between men’s and women’s experiences of loneliness. Such testing across genders is regularly carried out in connection with the development of psychometric instruments [66, 67]. Researchers have previously tested the measurement invariance across genders of various loneliness scales, such as the De Jong Gierveld loneliness scale [68] and the Loneliness and Aloneness Scale for Children and Adolescents [69]. Similarly, some studies have examined whether the UCLA Loneliness Scale has the same structure across men and women. Allen and colleagues used a short 7-item version of the UCLA Loneliness Scale [70] and found support for a unidimensional structure, which did not meaningfully differ between men and women. Hawkley and colleagues found support for a three-factor structure in both genders [40], using the 1980 [21] version of the UCLA. Finally, a study that was based on a sample of over 1,000 teachers in Canada and that used the second version of the full 20-item UCLA scale found support for a three-factor structure that was invariant between men and women [71]. To our knowledge, however, measurement invariance based on gender has not been established in a representative sample of the population, nor in a sample of older adults for the UCLA Version 3.

Our research contributes to the literature by examining measurement invariance of the UCLA Version 3 loneliness scale [22] in two separate samples: a UK-based adult online sample where participant age and gender were nationally representative (Sample 1), and an online sample of UK-based older adults (Sample 2). We examine one, two and three factor models via confirmatory factor analyses, and examine if we can establish whether this factorial structure is equivalent in men and women across our two different samples.

## Methods

Both studies were advertised on Prolific, a crowd sourcing website for scientific studies [72]. In a comparison of online platforms for recruiting participants, participants from Prolific failed fewer attention checks, showed lower levels of dishonest behaviour and were more naive in relation to common psychological research materials, as compared to participants from Amazon MTurk [73]. Potential participants are recruited to Prolific primarily via word-of-mouth (including on social media), following an original recruitment drive when Prolific was founded in 2014, which recruited via social media, flyer distribution at university campuses, and a paid refer-a-friend scheme [74]. Once signed up to the Profiific platform, participants have the opportunity to take part in research in exchange for monetary payment.

### Sample 1 (nationally representative adults)

We used the Prolific settings to request a sample of 500 UK-based adults whose age and gender were nationally representative. We obtained 498 complete responses (self-reported gender: 257 women, 236 men, 2 neither, 3 non-disclosures). Three participants did not provide their age, but for the remaining participants, the ages ranged from 19 to 82 years (*M* = 49.15, *SD* = 15.53). 289 out of 498 participants indicated that they had completed at least a Bachelor level degree. Participants who did not report their gender as male or female were excluded from the further analyses, given that we wished to examine measurement equivalence between men and women. One participant did not complete all items and was excluded from the Structural Equation Models (SEM). Thus, the final sample consisted of 492 participants. Participants were paid £3.35 for completing the survey.

### Sample 2 (older adults)

We used the Prolific settings to request a sample of UK-based adults aged 65 years old or older. 290 participants (179 women and 111 men) completed the survey. One participant did not report their age, and one reported an improbable value (66,123). As we did not include age as a factor in any of the analysis, these two participants were retained in the final sample. For the participants who provided their ages, the range was from 64 to 86 years (*M* = 69.04, *SD* = 3.88). 146 out of 290 participants indicated that they had completed at least a Bachelor level degree. Participants were paid £2 for completing the survey.

### Procedure

For Sample 1 (nationally representative adults), the UCLA Loneliness Scale was administered as part of a larger online egocentric social network study [75, 76]. The full study protocol was preregistered on the Open Science Framework (OSF). In Sample 2 (older adults) the UCLA Loneliness Scale was collected as part of a larger study where participants completed multiple scales on health, psychological well-being, and friendships. The protocol is registered on the OSF. Both studies were approved by the Northumbria University Psychology Department Ethics Committee, and participants recorded their consent within the online survey.

### Materials

#### Loneliness

In both studies, participants completed the UCLA Loneliness Scale Version 3 [22]. This scale contains 20 items, where 11 of these refer to positively valenced feelings such as feeling part of a group of friends, and 9 of these refer to negatively valenced feelings such as feeling left out, and are conventionally reverse-scored. Participants are asked to respond on a 4-point scale, anchored at 1 = Never and 4 = Always. In version 2 of the UCLA Loneliness Scale [21] a different endpoint was used (4 = Often). It is unclear why this change happened, and correspondingly some papers have used the older anchor (e.g., [56, 71]). In our study, Sample 1 used the version 2 anchors (never / often) from [21], and Sample 2 used the version 3 anchors (never / always) from [22]. The negatively valenced items were not reverse-scored for SEM, as this is not necessary. This just implies that there will be negative correlations between a negatively valenced factor and (an)other factor(s) in two and three factor solutions, rather than a positive one (if we had reverse-scored).

### Data analysis

Our analyses consist of Confirmatory Factor Analyses (CFA) and group invariance testing [77]. While there is an active debate about sample sizes in CFA and the use of heuristics to determine sample sizes (e.g., [78, 79]), we note that our sample exceeds a common heuristic of N = 200 (e.g., [80]), and is in line with other studies (e.g., [53]). All the analyses were conducted in R 4.0.2 [81] and various R packages (e.g., [82–84]). Among these packages, we used ‘lavaan’ [85] to perform CFA, following the one-factor solution proposed by [22], the two-factor solution proposed by [29], and the three-factor solution proposed by [40] (see Table 1 and [56]). We also attempted Russell’s [22’s] bifactor model (as supported by [52–54]—see Introduction), where all items load on to a general loneliness factor, and in addition each item is allocated to a “positive items” or a “negative items” factor, but this did not give rise to a reliable solution, and is not discussed further in this paper. Next, we examined measurement invariance [67, 86–88]. The Open Science Framework provides free public access to all data, code, and analyses, as well as further analyses and fit metrics not reported in text (e.g., Standardized Root Mean Square Residual, SRMR).

**Table 1:** Descriptive statistics of questionnaire items (1-4 scale) (Russell, 1996), Sample 1 (nationally representative adults, n = 492).

Item	Mean	SD
1. I feel in tune with the people around me.	3.051	0.584
2. I lack companionship.	2.413	0.853
3. There is no one I can turn to.	2.172	0.931
4. I do not feel alone.	2.363	0.848
5. I feel part of a group of friends.	2.807	0.827
6. I have a lot in common with the people around me.	2.830	0.656
7. I am no longer close to anyone.	2.331	0.938
8. My interests and ideas are not shared by those around me.	2.552	0.801
9. I am an outgoing person.	2.947	0.739
10. There are people I feel close to.	2.933	0.676
11. I feel left out.	2.446	0.788
12. My social relationships are superficial.	2.394	0.816
13. No one really knows me well.	2.643	0.889
14. I feel isolated from others.	2.420	0.867
15. I can find companionship when I want it.	3.034	0.858
16. There are people who really understand me.	2.872	0.764
17. I am unhappy being so withdrawn.	2.653	0.857
18. People are around me but not with me.	2.677	0.724
19. There are people I can talk to.	3.203	0.793
20. There are people I can turn to.	3.185	0.834

## Results

### Descriptive statistics

Tables 1 and 2 show the descriptive statistics for all items for Sample 1 (nationally representative adults) and Sample 2 (older adults), respectively. These are the raw scores, i.e. not reverse-scored.

**Table 2:** Descriptive statistics of questionnaire items (1-4 scale) (Russell, 1996), Sample 2 (older adults, n = 290).

Item	Mean	SD
1. I feel in tune with the people around me.	3.303	0.669
2. I lack companionship.	2.193	0.943
3. There is no one I can turn to.	1.914	0.920
4. I do not feel alone.	2.090	0.926
5. I feel part of a group of friends.	2.924	0.953
6. I have a lot in common with the people around me.	2.955	0.853
7. I am no longer close to anyone.	1.821	0.935
8. My interests and ideas are not shared by those around me.	2.400	0.887
9. I am an outgoing person.	3.266	0.764
10. There are people I feel close to.	3.179	0.773
11. I feel left out.	2.186	0.868
12. My social relationships are superficial.	2.117	0.880
13. No one really knows me well.	2.293	0.930
14. I feel isolated from others.	1.976	0.913
15. I can find companionship when I want it.	3.079	0.943
16. There are people who really understand me.	2.990	0.886
17. I am unhappy being so withdrawn.	2.355	0.989
18. People are around me but not with me.	2.231	0.851
19. There are people I can talk to.	3.300	0.817
20. There are people I can turn to.	3.341	0.765

When using the scale as a unitary construct, the Cronbach *α*s for the respective samples were .95 (Sample 1, nationally representative adults, *M* = 2.26, *SD* = 0.56) and .94 (Sample 2, older adults, *M* = 2.02, *SD* = 0.60).

### Sample 1 (nationally representative adults): Confirmatory factor analyses

Fit indices indicated that a model with three factors proved the best fit (Comparative Fit Index, CFI = .883, Tucker-Lewis Index, TLI = .866, Root Mean Square Error of Approximation, RMSEA = .095). A single factor model proved to be a substantially worse fit to the data (CFI = .804, TLI = .781, RMSEA = .121), as did a two factor model (CFI = .849, TLI = .830, RMSEA = .107).

Measurement invariance modelling showed that the model that produced the lowest RMSEA = .089 (Table 3; ‘Mean,’ Model 5) was the one where the factor loadings, intercepts, residual variances and means were constrained to be equal across groups. There is some loss of fit in terms of CFI moving from configural to mean invariance, but it falls within the suggested -.01 change [89] or -.02 change [90]. We, therefore, conclude that the factor means can be considered equal between groups: i.e. there are no measurable mean differences between men and women as regards these three latent constructs.

**Table 3:** Measurement invariance summary: Sample 1 (nationally representative adults, n = 492).

	*χ* ^2^	df	Δ*χ*^2^	df	p	CFI	ΔCFI	RMSEA	ΔRMSEA	BIC	ΔBIC
Configural	1069.7	334	NA	NA	NA	0.883	NA	0.095	NA	18581.6	NA
Metric	1098.9	351	29.2	17	0.033	0.881	0.002	0.093	0.002	18505.4	76.2
Scalar	1129.7	368	30.9	17	0.021	0.878	0.002	0.092	0.001	18430.9	74.5
Residual	1150.6	388	20.9	20	0.406	0.878	0.000	0.089	0.002	18327.8	103.1
Mean	1159.3	391	8.8	3	0.033	0.877	0.001	0.089	0.000	18318.0	9.8

**Fig 1:**
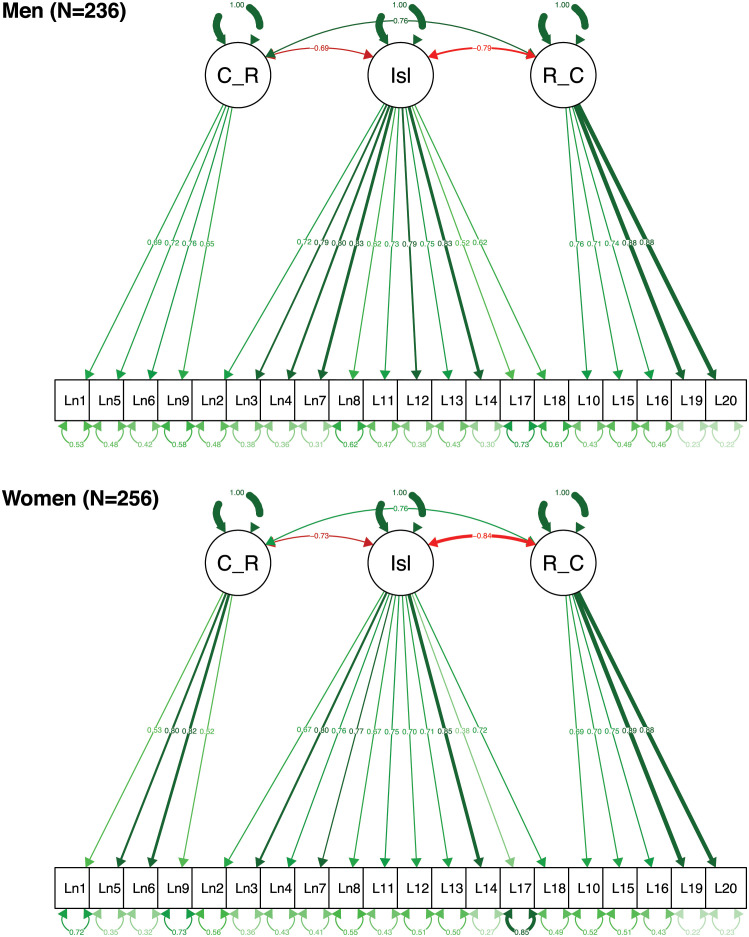
SEM plots for men and women from Sample 1 (nationally representative adults). C_R = Collective Relatedness; Isl = Isolation, R_C = Relational Connectedness. Note: Single headed arrows: factor loadings, double-headed arrows: covariances or error variances associated with items.

Fig 1 shows the resulting models for men and women. The labels are based on the model by Hawkley and colleagues [40]. The associations between the three latent constructs are also similar between men and women.

### Sample 2 (older adults): Confirmatory factor analyses

As in Sample 1, for Sample 2 fit indices indicated that a model with three factors proved the best fit (CFI = .873, TLI = .855, RMSEA = .100). It outperformed a two factor model (CFI = .833, TLI = .812, RMSEA = .113), which itself outperformed a single factor model (CFI = .757, TLI = .729, RMSEA = .136).

Measurement invariance modelling showed that the model where the factor loadings, intercepts, residual variances and means are constrained to be equal across groups produced the lowest RMSEA = .095 (Table 4; ‘Mean,’ Model 5). There is some loss of fit in terms of CFI moving from configural to mean invariance; it is close to the suggested -.01 change [89], but below the suggested -.02 change [90]. While the -.02 criterion is more liberal, on the whole Table 4 leads us to conclude that the factor means can be considered equal between groups, i.e. there are no measurable differences between men and women on these three latent constructs.

**Table 4:** Measurement invariance summary: Sample 2 (older adults, n = 290).

	*χ* ^2^	df	Δ*χ*^2^	df	p	CFI	ΔCFI	RMSEA	ΔRMSEA	BIC	ΔBIC
Configural	814.3	334	NA	NA	NA	0.873	NA	0.100	NA	12158.9	NA
Metric	847.0	351	32.7	17	0.012	0.868	0.004	0.099	0.001	12095.2	63.7
Scalar	869.0	368	22.0	17	0.184	0.867	0.001	0.097	0.002	12020.9	74.4
Residual	906.0	388	36.9	20	0.012	0.863	0.004	0.096	0.001	11944.4	76.5
Mean	908.0	391	2.0	3	0.571	0.863	0.000	0.095	0.000	11929.4	15.0

**Fig 2:**
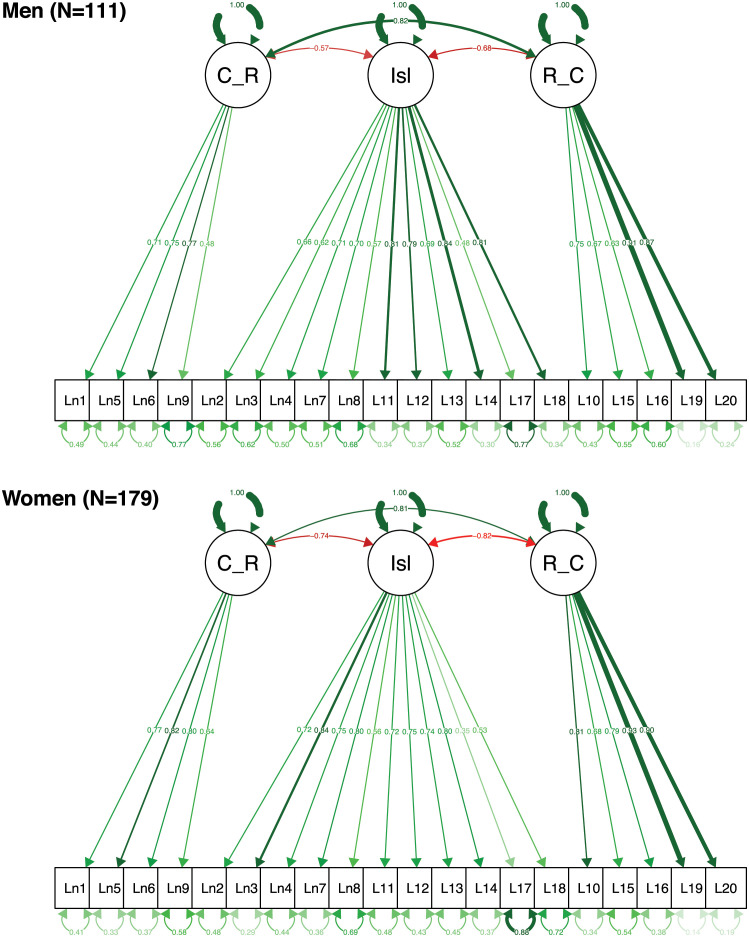
SEM plots for men and women from Sample 2 (older adults). C_R = Collective Relatedness; Isl = Isolation, R_C = Relational Connectedness. Note: Single headed arrows: factor loadings, double-headed arrows: covariances or error variances associated with items.

Fig 2 shows the resulting models for men and women in Sample 2 (older adults). The associations between the three constructs are also similar, as in Sample 1 (nationally representative adults). The only exception is that the association between Collective Relatedness and Isolation is somewhat lower in men (*r* = -.57) than in women (*r* = -.74) but the 95% confidence intervals still comfortably overlap (-.73 to -.41 and -.83 to -.66, respectively).

## Discussion

In this study, we investigated the factorial structure of the widely used UCLA Loneliness Scale for men and women in two different online samples: an adult UK sample that was nationally representative by age and gender, and a sample of UK older adults. In both samples, a model with three factors proved the best fit. Authors have reported slightly differing ways of allocating the 20 items of the UCLA Loneliness Scale (either the second or third version) to a three-factor structure model, and such solutions have been reported in several studies, including large samples from Argentina, Iran, Ireland, Spain, and Turkey ([52, 55, 56], see Introduction; but see [31] for contrasting results). We did not seek to test each of the slightly differing three-factor models in relation to our data to avoid over-fitting, but instead focussed on the popular Hawkley et al. [40] model (e.g., [56]). We also examined Russell’s [22] bifactor structure composed of three factors, but this model was not identified, see OSF. Our findings support the notion that the UCLA Loneliness Scale reflects loneliness as a multidimensional rather than a unidimensional structure, with three factors corresponding to Isolation (feelings of aloneness and rejection), Relational Connectedness (feelings of familiarity, closeness and support) and Collective Connectedness (feeling part of groups that provide a sense of identity and belonging), as suggested by [40, 56].

Prolonged periods of loneliness are consistently associated with poorer health outcomes [10], and as such tackling loneliness can be part of a country’s political and social agenda [91]. There are several different types of interventions to reduce loneliness [14, 15], including social prescribing approaches which are designed to provide a non-medical referral option for General Practitioner doctors to improve health and well-being [92]. In designing and evaluating these interventions, it is important to accurately measure the different facets of loneliness. For example, interventions that promote membership of community groups [92] may be more effective in providing a broader range of social connections (Collective Relatedness), as compared to emotionally close relationships (Relational Connectedness). As many interventions use the UCLA Loneliness Scale as an outcome measure [14, 15], if treated as a unitary scale this may miss these more subtle changes in different aspects of loneliness as a result of the intervention. Future work on loneliness should therefore consider treating the UCLA measure as a multidimensional measure, or use the other scales specifically designed to measure the different facets of loneliness (e.g., [93]).

The multidimensional nature of loneliness might reflect its differing etiologies, manifestations, and consequences, and thus might in turn be reflected across different questionnaire measures. As an example, the abbreviated Social and Emotional Loneliness Scale for Adults (SELSA) is also reported to have a three-factor structure [65]. Where the UCLA Loneliness Scale focuses perhaps more on the experience of loneliness, the SELSA focuses on its sources, and as such its subscales separate romantic, family, and social loneliness; for instance, an individual could have a strong relationship with a partner (romantic loneliness) and family (family loneliness), but not a strong friendship group (social loneliness). Previous research has shown relationships between people’s scores on the SELSA subscales and the UCLA [19, 93, 94], and we might anticipate further that the scores on the three UCLA factors would differentially predict scores on the SELSA subscales. For instance, we might predict particular overlap between the SELSA’s “social loneliness” and the UCLA’s “Collective Connectedness,” which incorporates items such as feeling part of a group of friends and feeling like you have a lot in common with the people around you. That is, loneliness, or the lack thereof, may depend on having both close and affiliative ties [32].

In addition to examining the overall factor structure of the UCLA scale, we also examined measurement invariance based on gender. We found support for the ‘means’ model in our analysis. This suggests that there are no meaningful differences between men and women in any of the three constructs. Now that we have established that the UCLA yields the same factorial structure for men and women, this enables researchers to make valid comparisons between men’s and women’s experiences of loneliness. Similarly, we note that the factor loadings, correlations, fit indices, and structure are similar across our two samples (nationally representative adults, and older adults), in line with [39].

Our samples were sourced from adults in the United Kingdom, and relied upon people who were enrolled on Prolific, a crowd-sourcing website for scientific studies. Thus, although our ‘nationally representative’ sample in Study 1 was representative in terms of age and gender, we would not expect them to be fully nationally representative of the United Kingdom, nor of course of other countries. Equally, adults aged 65 years old or older are less likely than other age groups to use the internet [95], and yet our ‘older adults’ sample all necessarily used the internet in order to access Prolific. It is important to be wary of assuming invariance in psychological variables across all countries and cultures [96, 97]. Having said this, we do not have serious concerns that our findings would be, prima facie, non-replicable in other samples. This is in part because other researchers report similar findings on the factor structure of the UCLA Loneliness scale in countries outside the UK (e.g., [52, 55, 56], but see [31]), and in part because of the affiliative and sociality requirements that are part of human nature [3], and that are indeed seen in related species [98].

In conclusion, we find support for a multidimensional (three-factor) structure to the UCLA Loneliness Scale, in a nationally-representative UK sample by age and gender, and in a UK sample of older adults. This multidimensional structure is consistent with previous research (e.g., [40, 56]), and is in line with the differing etiologies of loneliness (e.g., [32]). We suggest that our findings are broadly generalisable to other samples given the inherent sociality of humans as a species, although of course this awaits testing. We found no meaningful differences between men and women in any of the three constructs, something which supports the usage of the UCLA Loneliness Scale to compare men’s and women’s experiences of loneliness, and which may help us further tackle this important predictor of individual wellbeing (e.g., [10]). Future studies of loneliness should consider treating the UCLA Loneliness Scale as a multidimensional rather than unidimensional measure, or use other scales which are designed to measure the different facets of loneliness (e.g., [93]).
